# Study on the effect of pre-rehabilitation home-based on patients undergoing kidney transplantation with end-stage renal disease: A study protocol

**DOI:** 10.1097/MD.0000000000028280

**Published:** 2021-12-30

**Authors:** Xiaojie Ma, Zaozhang Zhang, Bonuan Yao, Mengsi Peng, Hongtao Jiang, Yong You

**Affiliations:** The Second Affiliated Hospital of Hainan Medical University, Haikou, China.

**Keywords:** end-stage renal disease, home exercise, kidney transplantation, pre-rehabilitation

## Abstract

**Objective:**

Evaluate the feasibility of ERAS-based home exercise pre-rehabilitation in patients awaiting kidney transplantation.

**Methodology:**

The proposed feasibility trial will be a single-arm, single-center study. A total of 47 ESRD patients awaiting kidney transplantation will be selected as subjects to undergo personalized family exercise pre-rehabilitation, including aerobic exercise, functional resistance exercise, and flexibility training. Briefly, a 6-minute walking test (6MWT), 4-meter gait speed, grip strength, and sit-to-stand test will be used as the main outcome indicators. The effect of family exercise pre-rehabilitation on the optimization of body function in ESRD patients undergoing kidney transplantation will be assessed. The days of hospitalization after kidney transplantation, postoperative complications, health survey (the Short Form Health Survey, SF-36), and the Hospital Anxiety and Depression Scale (HADS) will be used as secondary outcome indicators to evaluate the improvement of quality of life, psychological function, and postoperative rehabilitation of patients after kidney transplantation. These indexes will be collected before and after intervention (baseline and before kidney transplantation), before discharge (after kidney transplantation), and 1 month after discharge.

**Conclusion:**

This study will evaluate the effect of ERAS-based home exercise pre-rehabilitation on patients awaiting kidney transplantation, and possibly determine an application protocol for this population.

**Trial registration:**

Chinese Clinical Trial Registry, ChiCTR2000037846. Registered on September 2, 2020.

## 1 Introduction

End-stage renal disease (ESRD) refers to the development of chronic kidney disease to the end stage, the glomerular filtration rate is lower than 15 mL/(min 1.73 m^2^), and its treatment methods include hemodialysis, peritoneal dialysis, and kidney transplantation.^[1]^ According to the World Health Organization, the incidence of chronic kidney disease in China is higher than 10.8%, more than 0.03% of which will develop to ESRD,^[2]^ seriously affecting the life expectancy and quality of life of patients. Compared with dialysis treatment, renal transplant recipients have a higher survival rate and quality of life and can obtain better clinical outcomes.^[3]^ Kidney transplantation is the only radical treatment for ESRD. According to statistics, China has completed more than 10,000 kidney transplants in 2017.^[4]^ With the continuous maturity of kidney transplantation technology, optimizing perioperative management, reducing perioperative complications, and promoting rapid recovery of patients have become the focus of the transplantation team in recent years. The concept of surgery has changed from a “disease”-centered biomedical model to a “patient”-centered biomedical-psychological-social model, which promotes the continuous renewal of the concept of enhanced recovery after surgery (ERAS). ERAS adopts a series of perioperative optimization measures with evidence-based medical information. Moreover, ERAS implements a multi-disciplinary collaborative diagnosis and treatment model, such as surgery, anesthesia, nursing, nutrition, rehabilitation, and so on, to reduce the physical and psychological traumatic stress of surgical patients and promote the rapid recovery of patients.^[5]^ Internationally, the ERAS Association has registered in 2010. So far, 15 ERAS guidelines have been issued, including colorectal surgery, heart surgery, hip and knee replacement surgery, lung surgery, and so on.^[6]^ Although there are no guidelines related to ERAS of kidney transplantation, the research on the ERAS project of kidney transplantation has been reported.[^7^,^8^]

When waiting for kidney transplantation, ESRD patients will have functional disorders, such as frailty and decreased cardiopulmonary function, resulting in increased mortality and graft loss rate after kidney transplantation, which affects the clinical application compliance of ERAS regimen and increases the burden of medical insurance.^[9]^ Pre-rehabilitation can optimize the preoperative functional status of patients undergoing elective surgery and then the surgical prognosis.^[10]^ As a type of treatment scheme based on exercise, pre-rehabilitation aims to improve the health status of individuals before the operation to achieve better results after operation.^[11]^ It is an indispensable link in the concept of ERAS. At present, the most commonly used pre-rehabilitation management strategy based on the concept of ERAS is the new pre-rehabilitation management strategy (NEW), which includes nutritional (Nutrition), exercise (Exercise), and coping with anxiety (Worry).^[12]^ It has been confirmed in the intervention trials of cardiothoracic surgery and orthopedic surgery that pre-rehabilitation can significantly improve the short-term effects (hospital stay and postoperative complications) and long-term effects (functional ability and quality of life).^[13]^ In the related studies of abdominal surgery, exercise-based pre-rehabilitation for 3 to 8 weeks can improve the cardiopulmonary function of patients.^[10]^ ESRD patients awaiting kidney transplantation, due to functional decline and muscle atrophy, as well as the desire for kidney transplantation, expect to improve their health to increase the success rate of transplantation, reduce the incidence of postoperative complications, and improve their compliance with pre-rehabilitation, which is very suitable for pre-rehabilitation plans.

In addition to living donation, the timing of kidney transplantation in ESRD patients is unpredictable, and the timing of pre-rehabilitation remains uncertain. The compliance of early implementation will be poor, while patients will not benefit from a too late implementation either. During the period of waiting, ESRD patients need regular hemodialysis or peritoneal dialysis treatment. Regular outpatient exercise increases the time and financial burden of ESRD patients, especially those undergoing peritoneal dialysis. Studies have shown that patients prefer family- and community-based exercise during hemodialysis in hospitals.^[14]^ Therefore, pre-rehabilitation exercise programs conducted at home may be ideal and can reduce the time and financial burden caused by regular visits to rehabilitation clinics. In the study on patients undergoing coronary artery bypass grafting and heart valve surgery, Waite et al have found that preoperative family pre-rehabilitation can reduce the debilitating state of patients, improve the physical function, and shorten the length of hospital stay.^[15]^ This finding shows that family pre-rehabilitation has the potential in improving function and shortening hospitalization days. Williams and others have also actively explored the application of family exercise in patients awaiting liver transplantation.^[16]^ However, the application in ESRD patients awaiting kidney transplantation lacks detailed reports at home and abroad, and more research is necessary to explore its evidence-based medicine.

We hypothesized that personalized family exercise programs for ESRD patients can optimize functional ability, confidence, and motivation during waiting before kidney transplantation, reduce postoperative complications, and optimize transplant results. In the present study, we aim to optimize the family exercise training on the functional ability of ESRD patients undergoing kidney transplantation, and to reduce the adverse effects of frailty, decreased cardiopulmonary function, and psychological dysfunction on the prognosis of kidney transplantation. Before a randomized control trial (RCT) can be conducted, a feasibility study is required to determine whether a larger trial is possible and if so the optimally designed features should be outlined. Therefore, we will conduct a single-center feasibility trial of a novel home-based exercise program in ERSD patients awaiting kidney transplantation. We expect to provide a scientific and valuable reference for the application of family exercise pre-rehabilitation in patients awaiting kidney transplantation.

## 2 Materials and methods

**Figure 1:**
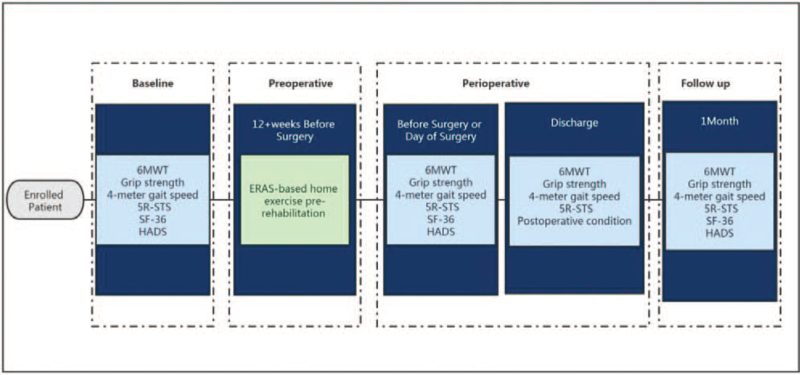
Study schema.

The proposed feasibility trial is a single-arm, single-center study of an ERAS-based home exercise pre-rehabilitation for patients listed for kidney transplantation. ESRD patients awaiting kidney transplantation will be selected as subjects to undergo personalized family exercise pre-rehabilitation, including aerobic exercise, functional resistance exercise, and flexibility training. Briefly, a 6-minute walking test (6MWT), 4-meter gait speed, grip strength, and sit-to-stand test will be used as the main outcome indicators. The effect of family exercise pre-rehabilitation on the optimization of body function in ESRD patients undergoing kidney transplantation will be assessed. The days of hospitalization after kidney transplantation, postoperative complications, health survey (the Short Form Health Survey, SF-36), and the Hospital Anxiety and Depression Scale (HADS) will be used as secondary outcome indicators to evaluate the improvement of quality of life, psychological function, and postoperative rehabilitation of patients after kidney transplantation. These indexes will be collected before and after intervention (baseline and before kidney transplantation), before discharge (after kidney transplantation), and 1 month after discharge. In addition, age, sex, race, type of occupation, marital status, education, body mass index (BMI), duration of dialysis, causes of ESRD, and types of renal replacement therapy should be recorded. A study schema is provided in Figure 1.

### 2.1 Setting

The study will be carried out at the Organ Transplant Center of the Second Affiliated Hospital of Hainan Medical University, a 2000-bed Grade 3A general hospital affiliated to Hainan Medical College. This hospital has obtained the national qualification for kidney transplantation in 2010, with annual kidney transplants of about 150.

### 2.2 Study registration

The trial protocol is approved by the institutional review board in the Second Affiliated Hospital of Hainan Medical University (LW2020028) and registered on September 2, 2020 at the Chinese Clinical Trial Registry (http://www.chictr.org.cn/showproj.aspx?proj=58570): ChiCTR2000037846.

### 2.3 Inclusion criteria

The inclusion criteria will be set as follows:

1. ESRD patients are estimated to undergo kidney transplantation in more than 12 weeks, and the first kidney transplant is on the waiting list of the deceased donor in the Second Affiliated Hospital of Hainan Medical University;
2. the age is 18 to 60 years old; and
3. patients volunteer and sign the informed consent form.

### 2.4 Exclusion criteria

The following patients will be excluded from the study:

1. patients with unstable angina pectoris, new arrhythmia, recent myocardial infarction, or unstable cardiovascular events;
2. patients with a recent cerebrovascular accident, or a modified RANKIN score (Modified Rankin Scale, mRS) ≥ 3; and
3. those who cannot participate in the exercise due to other serious diseases.

### 2.5 Study intervention

All subjects will complete all baseline assessments by trained physiotherapists, who work out and demonstrate personalized exercise prescriptions for each patient according to the results of the functional evaluation, including aerobic exercise, resistance training, and flexibility training. Patients will be trained to correctly use self-perceived exertion (Borg) to monitor exercise intensity, distribute family exercise diaries, and exercise bracelets. In addition to dialysis treatment while waiting for kidney transplantation, the family exercise pre-rehabilitation needs to be completed until the patient returns to the hospital for kidney transplantation, and the functional ability, quality of life, and psychological function of all participants will be then re-evaluated. If the participants stop exercising for 1 week or several weeks due to illness, they can participate intermittently and record the disease and intermittent participation in the case report table, which will be explained in the data analysis.

#### 2.5.1 Aerobic exercise prescription

Table 1 provides an overview of the aerobic exercise prescription.

**Table 1:** Aerobic exercise prescription.

Type	Walk
Intensity	Moderate intensity (40%–60% of the heart rate reserve value), calculated according to the results of resting heart rate, maximum heart rate, and distance in the 6MWT, the walking distance is given to the subjects, and the self-perceived exertion method (Borg11–13) is used to monitor the intensity during exercise.For example, in the 6MWT, the resting heart rate is 65 beats/min, the maximum heart rate is 95 beats/min, and the distance is 500 m.Target heart rate: (maximum heart rate-resting heart rate) ∗ 60% + resting heart rate = 83 (±5) beats/min, borg fatigue score is 11–13, 500X60%X5 = 2125. It is recommended that the patient should undergo a 30-min walk of 2125 m.
Time	Walk continuously or intermittently for 40 min.(including 5 min of warm-up, 30 min of exercise, and 5 min of finishing)
Frequency	Five times a week

#### 2.5.2 Functional resistance training

**Figure 2:**
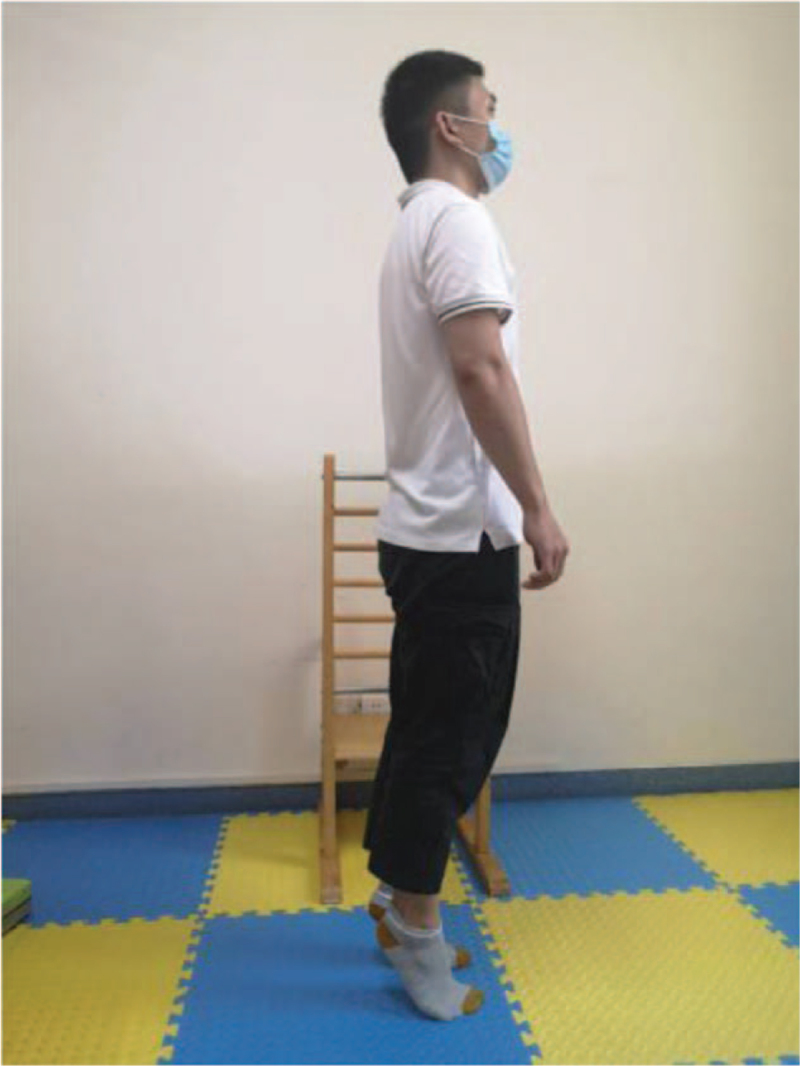
Heel up.

**Figure 3:**
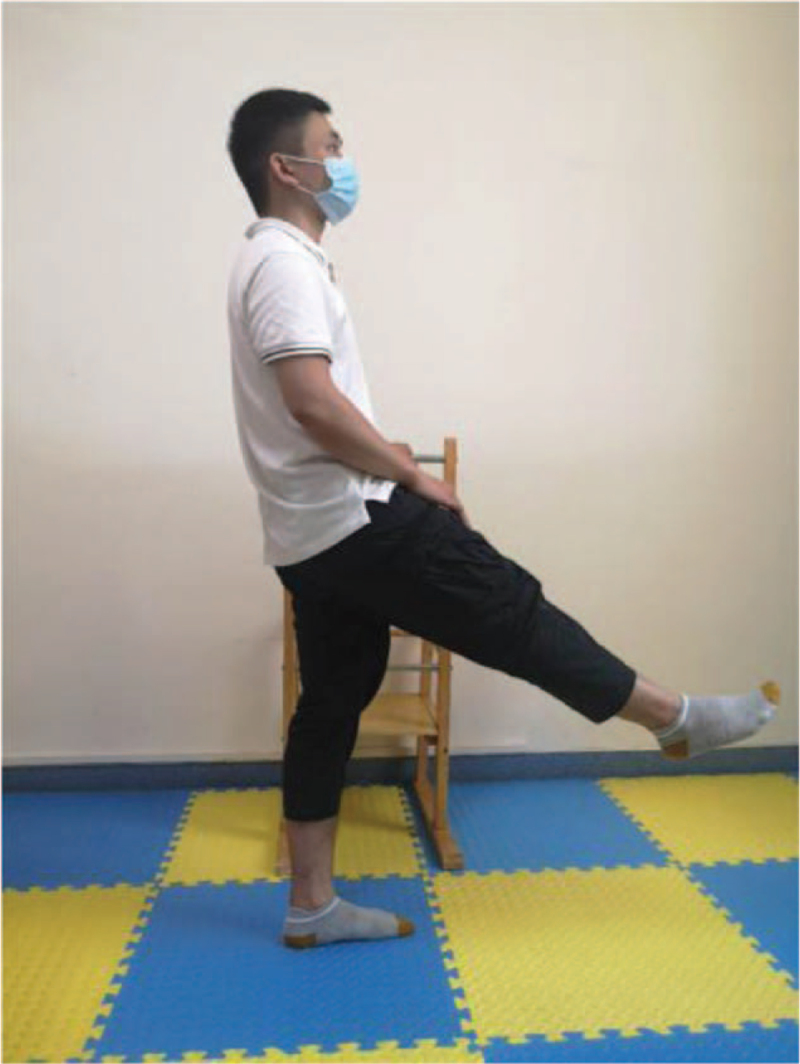
Kick.

**Figure 4:**
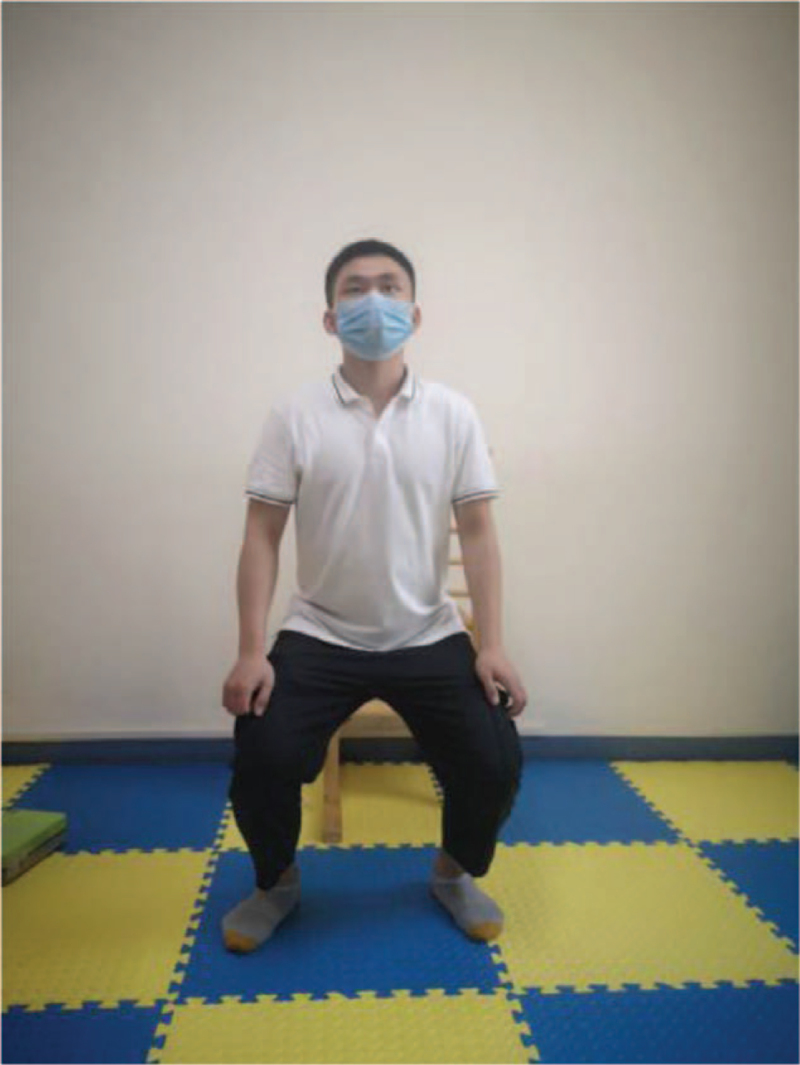
Squat up.

**Figure 5:**
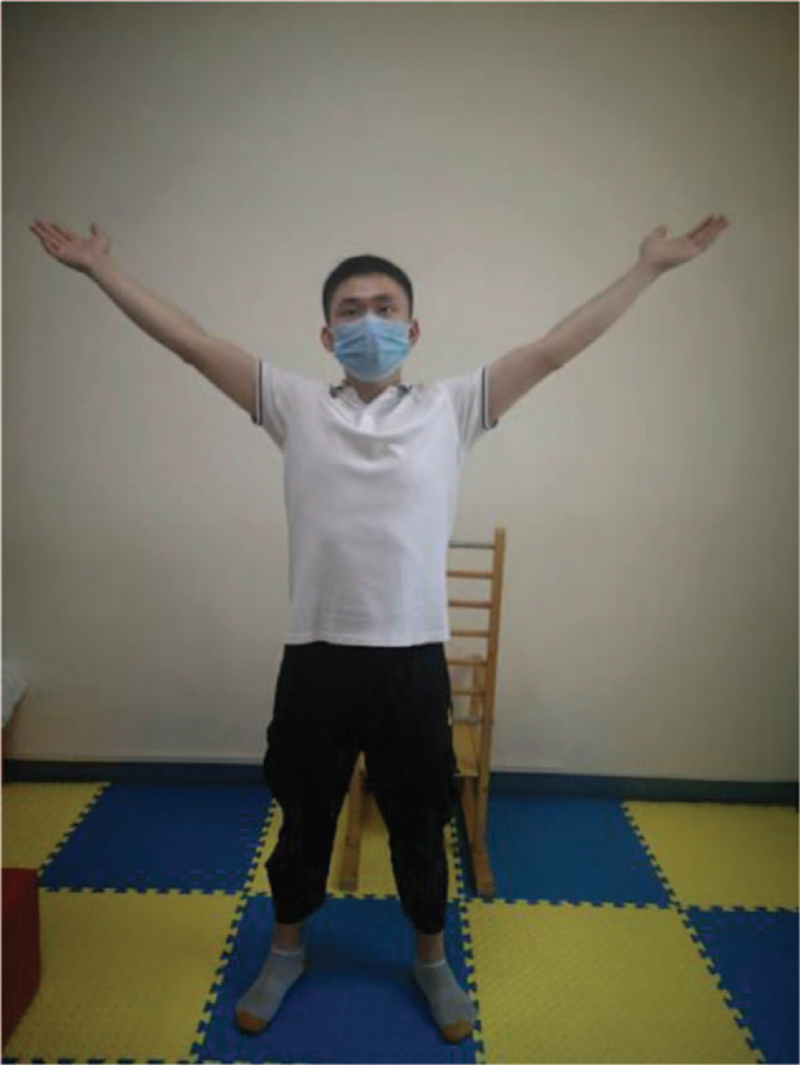
Dilatation of chest.

**Figure 6:**
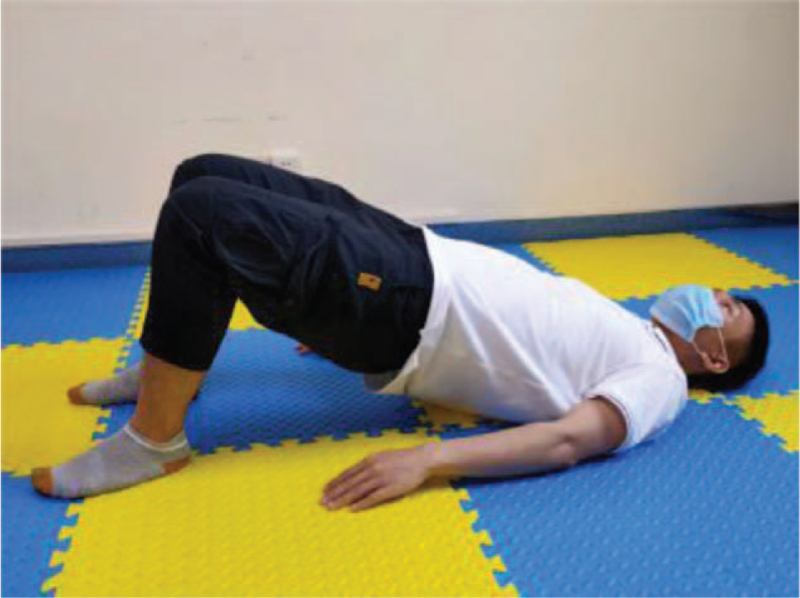
Bridge movement.

**Figure 7:**
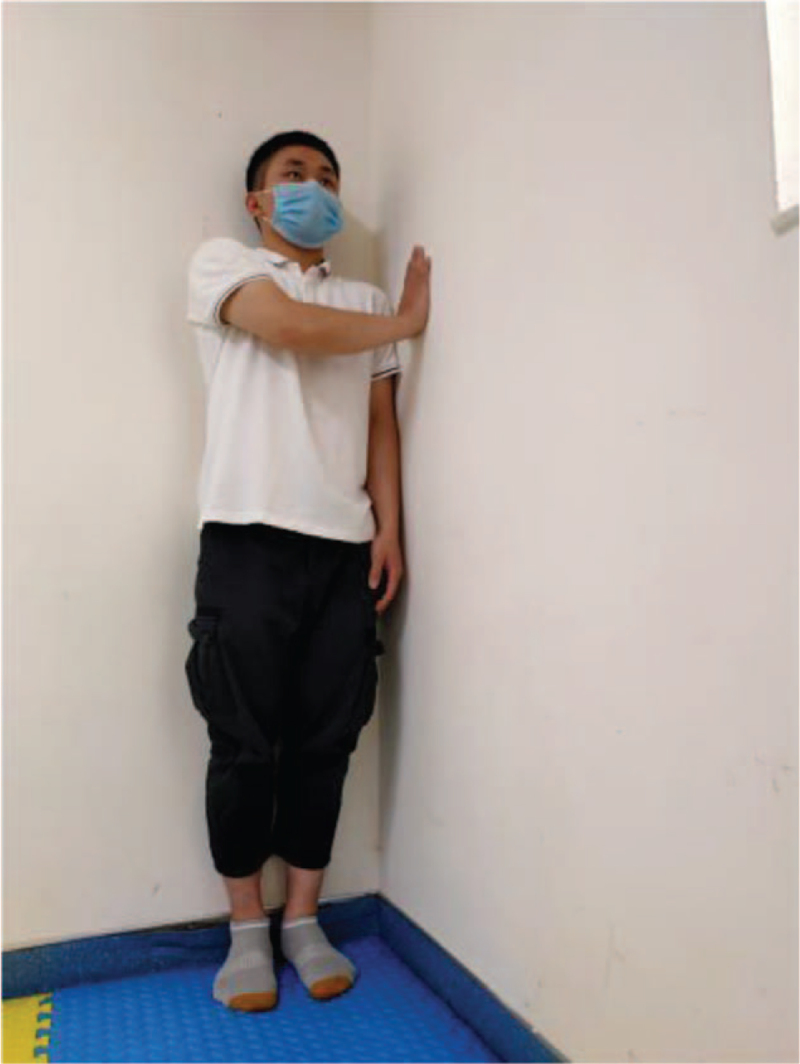
Push the wall.

According to the weakness of lower limbs in ESRD patients, as well as the damage of external oblique muscle, internal oblique muscle, transverse muscle, and rectus abdominis muscle caused by Rutherford Morison incision and Alexandra incision in common operation methods of kidney transplantation, the following functional training is designed to strengthen the muscle strength reserve (Table 2). This process will be combined with breathing training at the same time, exhale when exerting, and inhale when relaxing, and each action is repeated 8 to 12 times. The training intensity is defined by self-perceived exertion (Borg11–13), and the frequency is 3 times a week (Figs. 2–7).

**Table 2:** Functional resistance training.

Training action	Action diagram	Action points
Heel up	Figure 2	Hold the object by hand, keep your legs as wide as your shoulders, exhale and slowly lift your heels to the highest point to maintain 3–5 s (calm breathing here), then exhale and slowly land on your heels to relax.
Kick	Figure 3	Hold the object by hand, keep the legs as wide as the shoulder, one side of the lower limb bend the hip and knees, then keep the hip flexion and straighten the knee, and then extend the entire lower limb. Repeat this action alternately from left to right.
Squat up	Figure 4	Hold the object by hand, feet apart, shoulder wide, exhale with knee flexion not exceeding toe, the center of gravity sinking, maintain 3–5 s (calm breathing here), exhale and stand up and relax.
Dilatation of chest	Figure 5	Keep your arms straight, gradually raise your head forward and upward, and inhale deeply at the same time; then retract your arms and exhale deeply at the same time.
Bridge movement	Figure 6	Take a supine position, hands flat on both sides of the body, legs flexion, feet flat on the bed, arms lifted off the bed, maintain 3–5 s (calm breathing here), exhale, and relax.
Push the wall	Figure 7	Stand in the corner of the wall, back against one wall, push the other wall with the outside hand, maintain 3–5 s (breathe calmly here), relax and proceed alternately from side to side.

#### 2.5.3 Flexibility training

**Figure 8:**
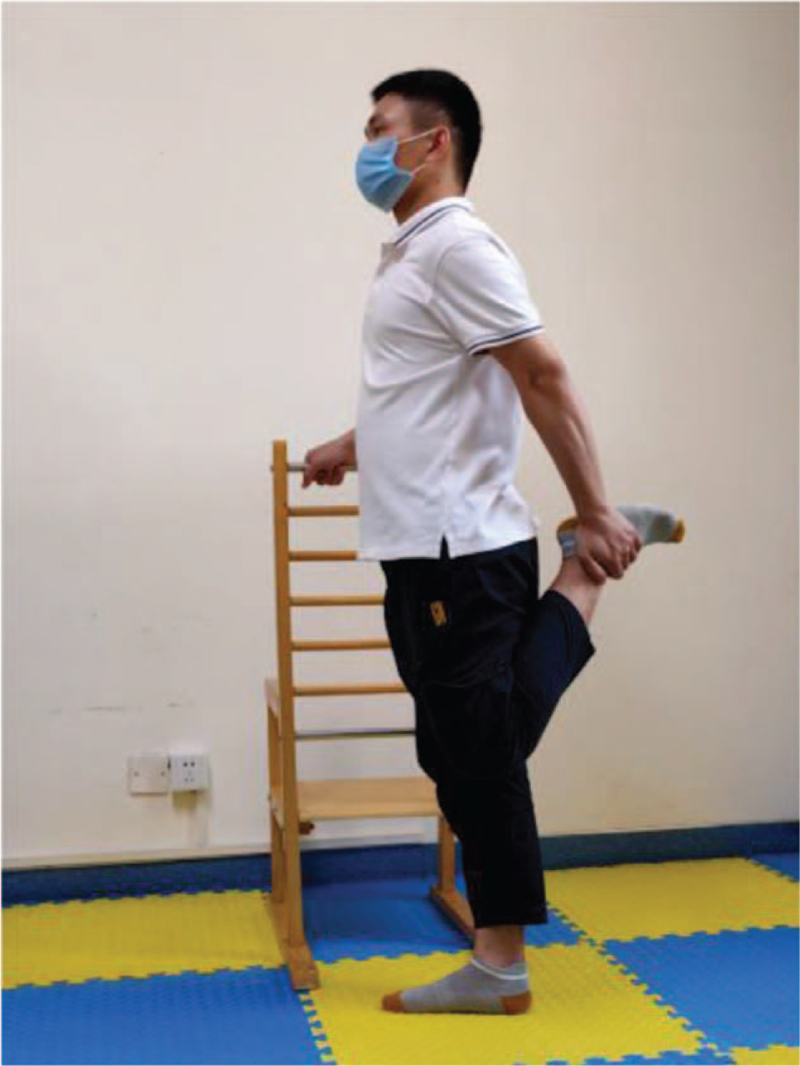
Stretch the front of the leg.

**Figure 9:**
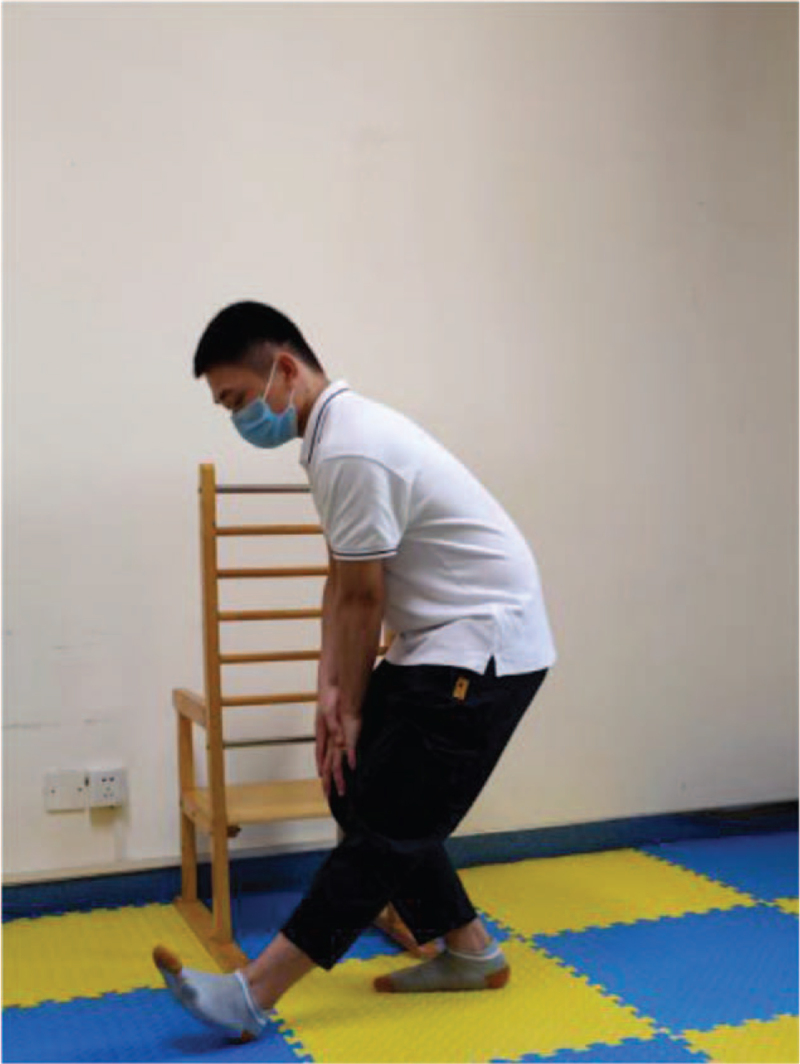
Stretch the back of the leg.

**Figure 10:**
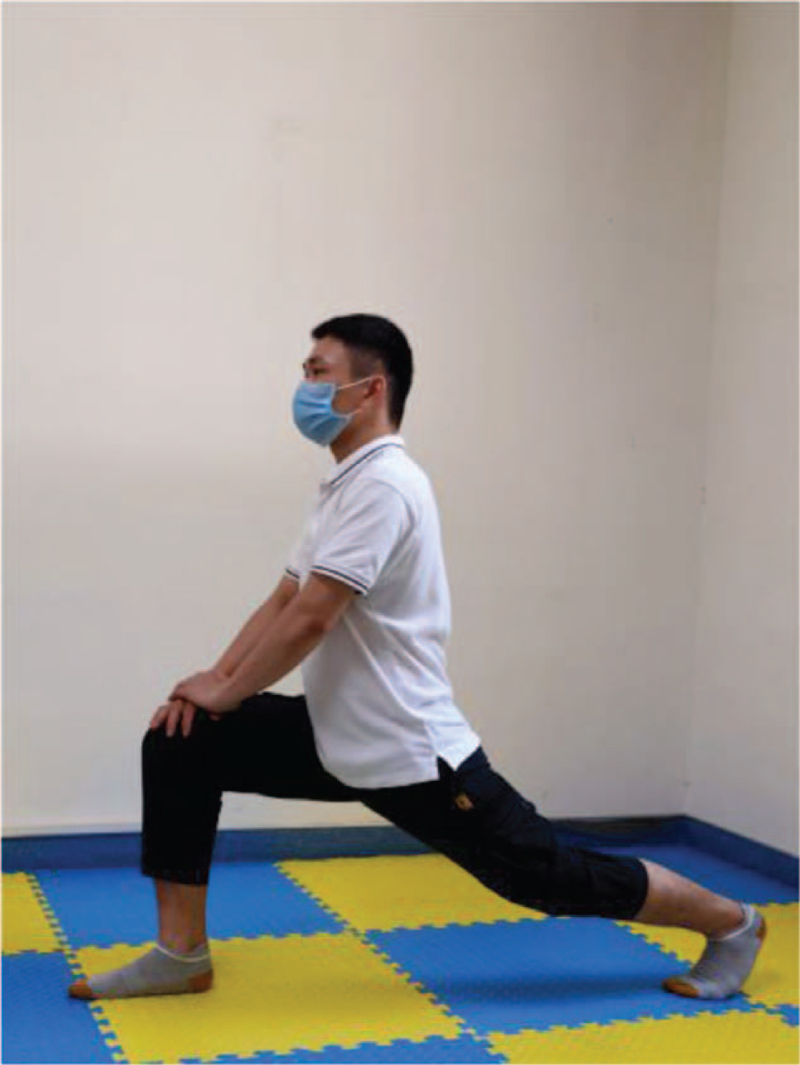
Calf stretch.

After the end of aerobic exercise and resistance training, the stretch will be carried out for each resistance training muscle group (Table 3), the duration of each action is 20 s, which is repeated 2 to 3 times, the total time is 10 minutes, and even breathing is maintained during the whole course (Figs. 8–10).

**Table 3:** Flexibility training.

Training action	Action diagram	Action points
Stretch the front of the leg	Figure 8	Hold the object with your hands, stand on one leg, grab the left foot with your left hand and pull it toward your buttocks. Keep the upper body as straight as possible (feet as close to the buttocks as possible) to maintain 20 s, 30 s, left, and right alternately.
Stretch the back of the leg	Figure 9	Bend your right leg, hold your left knee with both hands, straighten your left leg and hook your toes, bend your waist and back straight down, and maintain 20 s, 30 s, left, and right alternately.
Calf stretch	Figure 10	The right leg in front of the bow and arrow, the left toe forward, the body to do forward, downward elastic stretch, the left heel does not leave the ground, the total process 20–30 s.

### 2.6 Study outcomes

#### 2.6.1 Primary outcomes

1. Cardiopulmonary function: the cardiopulmonary function will be evaluated by 6MWT, and the maximum distance that the subjects can reach will be measured. The 6MWT is significantly correlated with peak oxygen consumption,^[17]^ which has good reliability and validity and can predict the adverse outcome of ESRD.^[18]^
2. Physical fitness: physical fitness will be evaluated by grip strength, 4-meter gait speed and 5 chairs repeated standing tests (5R-STS). Grip strength and lower limb muscle strength are good indicators of systemic muscle strength and reliable predictors of increased risk of hospitalization and death.^[19]^

#### 2.6.2 Secondary outcomes

1. Postoperative condition: the occurrence probability of postoperative complications, such as hospital stay, postoperative pulmonary infection and bleeding, postoperative drainage, and extubation time is recorded.
2. Quality of life: the quality of life is evaluated by the SF-36, which includes physical function, limitation caused by the body, body pain, general health, vitality, social function, limitation caused by emotion, and mental health. The higher the comprehensive score, the better the quality of life.^[20]^
3. Psychological evaluation: the HADS will be used to evaluate the emotional state of anxiety and depression. There are several problems of anxiety and depression respectively. The higher the total score, the more serious the anxiety or depression. This scale has good reliability and validity in our general hospitals.^[21]^

### 2.7 Data collection

In addition to the data collection involving our primary and secondary outcomes, the information regarding patient demographics and clinical variables, such as age, sex, type of occupation, marital status, education, BMI, dialysis of history, and frequency, causes of ESRD, and type of renal replacement therapy, will be also collected. The above-mentioned features are selectively collected on the admission day (baseline), on the day before operation (second follow-up), on the discharge day (third follow-up), and 4 weeks post-operation (fourth follow-up). The proportion of patients who agree to participate in the trial and the reasons for disagreement will be summarized. Moreover, the number and percentage of participants who complete the complete exercise plan will be also recorded. Scheduled data collection at each time point is presented in Table 4.

**Table 4:** Scheduled events and timeline of the trial.

	Study period					
	Time point	Enrollment	Preoperative	perioperative		Follow up
		12+ weeks before surgery	Before surgery or day of surgery	Discharge	1 month	
Enrollment	**Eligibility screening**	×				
	Informed consent	×				
Interventions	ERAS-based home exercise pre-rehabilitation		×			
Assessments	6MWT	×		×	×	×
	Grip strength	×		×	×	×
	4-meter gait speed	×		×	×	×
	5R-STS	×		×	×	×
	Postoperative condition				×	
	SF-36	×		×		×
	HADS	×		×		×

### 2.8 Compliance management

Given a home-based setup, it is important to promote adherence and conduct compliance management. To promote adherence to the program, a self-reported sports diary and a sports bracelet will be given to each participant to provide daily visual feedback and empower responsibility for their daily and weekly goals. Additionally, following a demonstration of the functional resistance exercises at their initial assessment, participants are provided with written and pictorial instructions, as well as a video of all of the exercises. All subjects are pulled into a WeChat group, each exercise record is uploaded to the group, and the “social comparison theory” is adopted to promote patients to complete the exercise.^[22]^ In addition to encouraging patients to complete exercise and punch on time at WeChat Group, the experimenter will conduct weekly telephone surveys to encourage patients to complete family exercise diaries. Any problem can be reported, such as pain or discomfort, to adjust the exercise prescription appropriately. The patients are encouraged during telephone counseling, and their efforts are praised.

### 2.9 Satisfaction survey

Before the patient is discharged from the hospital after the kidney transplantation, the satisfaction survey will be completed (Table 5), and the patient is asked to make suggestions on the family exercise program.

**Table 5:** Satisfaction survey.

Satisfaction survey	
Satisfaction with home exercise pre-rehabilitation	□Very dissatisfied □Dissatisfied □Generally □satisfied □ very satisfied.
Does home exercise pre-rehabilitation help you?	□Very dissatisfied □Dissatisfied □Generally □satisfied □ very satisfied.
The degree of difficulty of home exercise pre-rehabilitation	□Very difficult □difficult □Generally □Easy □Very easy

### 2.10 Sample size calculation

According to existing literature (Manfredini Fabio, Mallamaci Francesca, D’Arrigo Graziella et al. Exercise in Patients on Dialysis: A Multicenter, Randomized Clinical Trial.[J]. J Am Soc Nephrol, 2017, 28: 1259–1268), G-power3.1 software will be used to calculate the sample size. By paired-samples *t* test, the effect size = 0.37, α = 0.05, and power size = 0.80, and the calculated sample size is 47.

### 2.11 Statistical analysis

SPSS24.0 statistical software and Office Excel 2016 will be used to analyze the test data. The quantitative data will be tested by the Kolmogorov–Smirnov test to determine whether they are following a normal distribution. Quantitative data: if the comparison before and after the intervention follows the normal distribution, the paired sample *t* test will be used; if it does not meet the normal distribution, the non-parametric test Wilcoxon symbolic rank test will be used for analysis. The results are expressed as mean ± standard deviation ( $\bar{X}\pm S$ ). The postoperative hospitalization days, postoperative complications, postoperative drainage, and extubation are compared with the previous data in the hospital. If the data satisfies normal distribution, an independent sample *t* test will be used; otherwise, the Mann–Whitney *U* test is used for analysis, and the results are all expressed as mean ± standard deviation ( $\bar{X}\pm S$ ). The qualitative data will be analyzed by chi-square test (χ^2^ test), and the results are expressed as a percentage (%). The level of significance is *P* < .05.

### 2.12 Adverse events and analysis

An adverse events (AEs) will be reported at initial screening and continue until 30 days post each participant's kidney transplantation. The symptom degree, occurrence time, duration, and treatment of AE should be recorded in the observation table, and the correlation between AEs and clinical intervention training should be evaluated based on comprehensive consideration and recorded in detail by managers. The researchers can suspend the intervention, adjust the training cycle, deal with it according to the situation, and report it to the ethics committee of the research institution and the research unit if necessary to closely observe the outcomes of the event.

## 3 Discussion

With the increasing number of renal transplant operations, the continuous maturity of surgical methods, and the pursuit of better clinical outcomes, people's interest in the ERAS program has greatly increased. Pre-rehabilitation before operation can improve the baseline function of patients, enhance surgical tolerance, and reduce postoperative complications.

When waiting for renal transplantation, ESRD patients often show frailty and decreased cardiopulmonary function, leading to increased complications and mortality after renal transplantation. Preoperative pre-rehabilitation can optimize the health status of individuals before the operation. Therefore, patients can achieve better results after operation. The family based pre-rehabilitation exercise program has been proved to be effective in heart surgery, liver transplantation, and other related fields. The family sports plan is simple, and the subjects are easy to grasp the essentials of their movements, including walking, strength training, and other sports, which do not need professional equipment and are not affected by the venue, weather, and other factors. Pre-rehabilitation exercise programs for renal transplantation in the family can reduce the time and economic burden brought by dialysis patients going to the rehabilitation clinic regularly. In the present trial, we, for the first time, will assess an ERAS-based home exercise pre-rehabilitation in patients listed for kidney transplantation.

In the present study, participants will record their activities in their sports diaries and report them in WeChat groups. In a larger RCT, real-time data collection can be considered. Physiotherapists can monitor the compliance and progress of the exercise program every day and avoid reporting bias by participants.

Both objective and subjective parameters will be included in the primary and secondary outcomes. We should not only pay attention to the objective incidence of hospital stay and postoperative complications but also consider the impact on the patient-based HRQoL. Patients can return to family and society in the best state by improving HRQoL, embodying the core of diagnosis and treatment in the concept of ERAS, which is centered on serving patients.

This Phase I trial is essential to understand potential recruitment rates, dropout rates, patients who receive transplants or die during the study, and the completion rate of this program to accurately motivate participants for future RCTs.

Collectively, this trial is a practical, single-center, single-arm, exploratory clinical trial. We plan to show that ERAS-based home exercise pre-rehabilitation will reduce the adverse effects of frailty, cardiopulmonary dysfunction, and psychological dysfunction on the prognosis of renal transplantation.

## 4 Conclusions

This study will evaluate the effect of ERAS-based home exercise pre-rehabilitation on patients awaiting kidney transplantation, and possibly determine an application protocol for this population.

## Author contributions

Conceptualisation, X.M.,Z.Z. and Y.Y.; methodology, X.M., Z.Z.,Y.Y., M.P., B.Y.,H.J; writing-original draft preparation, X.M., Z.Z.,Y.Y.; data curation, X.M.,Z.Z., M.P., B.Y, Z.Z.; writing-review and editing, X.M., Z.Z.,Y.Y; funding acquisition, X.M. All authors have read and agreed to the published version of the manuscript.

**Conceptualization:** Xiaojie Ma, Zaozhang Zhang, Yong You.

**Data curation:** Xiaojie Ma, Zaozhang Zhang, Bonuan Yao, Mengsi Peng.

**Funding acquisition:** Xiaojie Ma.

**Methodology:** Xiaojie Ma, Zaozhang Zhang, Bonuan Yao, Mengsi Peng, Hongtao Jiang, Yong You.

**Writing – original draft:** Xiaojie Ma, Zaozhang Zhang, Yong You.

**Writing – review & editing:** Xiaojie Ma, Zaozhang Zhang, Yong You.
